# Cross-cultural generalizability of suicide first aid actions: an analysis of agreement across expert consensus studies from a range of countries and cultures

**DOI:** 10.1186/s12888-018-1636-8

**Published:** 2018-03-01

**Authors:** Anthony F. Jorm, Anna M. Ross, Erminia Colucci

**Affiliations:** 1 Centre for Mental Health, Melbourne School of Population and Global Health, The University of Melbourne, 207 Bouverie St, Carlton, VIC 3010 Australia; 2 Department of Psychology, Middlesex University London, London, UK

**Keywords:** Suicide, Mental health first aid, Delphi studies, Guidelines, Gatekeepers

## Abstract

**Background:**

A number of Delphi expert consensus studies have been carried out with different countries and cultural groups to develop guidelines on how a member of the public should provide assistance to a person who is suicidal. The present study aimed to determine whether cross-culturally generalizable suicide first aid actions are possible by comparing agreement across these Delphi studies.

**Methods:**

Data on endorsement rates for items were compared across six Delphi studies. These studies involved panels of professionals and consumer advocates from English-speaking countries, professionals from Sri Lanka, professionals from Japan, professionals from India, professionals from the Philippines, and professionals and consumer advocates in refugee and immigrant mental health. Correlations were calculated between item endorsement rates across panels.

**Results:**

There were 18 items that were highly endorsed across all eight of the Delphi panels and an additional 15 items highly endorsed across the panels from the three lower middle-income countries (India, Philippines and Sri Lanka). Correlations across panels in item endorsement rates were all 0.60 or above, but were higher between panels from countries that are socioeconomically similar.

**Conclusions:**

There is broad agreement across the diverse expert panels about what are appropriate suicide first aid actions for members of the public, indicating that cross-cultural generalizability is possible. However, there is also some cultural specificity, indicating the need for local tailoring.

## Background

Individuals in a suicidal person’s social network have the potential to take action that may reduce the risk of suicide. Because it is not feasible to carry out trials in which family and friends are randomly allocated to carry out specific actions to assist a suicidal person, expert consensus has been used to determine what are likely to be helpful actions in this area. This approach involves systematically gathering ‘practice-based evidence’ using the Delphi method, based on experts’ professional and/or personal experience [1]. It accords with ‘wisdom of crowds’ research showing that, under certain conditions, groups of individuals who each have imperfect expertise can make good decisions when their expertise is aggregated [1].

In the initial Delphi study on this topic, Kelly and colleagues [2] did a systematic search of advice on how to assist a suicidal person and used this to construct a questionnaire of 114 statements. This questionnaire was presented to a panel of 22 professionals and 16 people with lived experience (they had been suicidal or had cared for someone who had been suicidal in the past) who were from developed English-speaking countries. There were 30 items which were endorsed at a high level by both panels and these were used to produce guidelines on mental health first aid for suicidal ideation and behavior. These guidelines were subsequently used to inform the content of a Mental Health First Aid training course which has been widely disseminated [3]. The guidelines were also made available for free download online, and a survey of people who downloaded them found that some users made positive use of the guidelines to assist a suicidal person [4].

A new Delphi study was later carried out in order to update these guidelines [5]. A systematic search led to development of a questionnaire with 436 items about knowledge or actions to assist a suicidal person. These items were rated by panels of 42 suicide prevention professionals and 35 consumer advocates from developed English-speaking countries, and 164 items were endorsed at a high level. The items cover identification of suicide risk, assessing the seriousness of risk, initial assistance, talking with the suicidal person, no-suicide contracts, what the first aider should know, confidentiality and adolescent-specific considerations. The resulting updated mental health first aid guidelines for suicidal thoughts and behaviours were used to revise the content of Mental Health First Aid training [6–9]. More recently, the guidelines have been used as the basis of a special training course on Mental Health First Aid for the Suicidal Person [10]. These guidelines for the public differ from clinical practice guidelines for general practitioners and psychiatrists in that they do not involve formal assessment of clinical state, diagnostic labels or treatment plans [11, 12].

Because there may be cultural factors that affect provision of assistance by family, friends and other helpers [13], the Delphi studies on suicide first aid [2, 5] only used experts from developed English-speaking countries. However, the authors cautioned that: “The application of the guidelines to non-western cultures and ethnic minorities is an area requiring further investigation and consultation with suicide prevention experts from these cultural and ethnic backgrounds” [5].

Because of the potential cultural differences in how to best assist a suicidal person, a series of Delphi studies has also been carried out with professional experts in a number of Asian countries, using the questionnaires of either of the English-speaking country Delphi studies as a starting point [2, 5], but supplemented with additional items relevant to the specific culture. These studies have been carried out for India [14], Japan [15], Philippines [16] and Sri Lanka [17]. Cultural differences may also be important for ethnic minority groups within countries [18, 19]. For this reason, a Delphi study has also been carried out on assisting suicidal persons from refugee and immigrant backgrounds [20].

While a culturally-specific approach to developing suicide first aid guidelines might be the ideal, it is resource intensive and would be a major task to implement for every potential cultural group in the world. The aim of the present study is to explore the feasibility of having cross-culturally generalizable suicide first aid guidelines. The study does this by comparing the endorsement rates of expert panels from the various existing Delphi studies to see whether there are common actions recommended across all the studies, and also across the three lower-middle income countries specifically. The study also quantifies the degree of agreement across expert panels from the various cultural groups.

## Methods

### Data compilation

Data from Delphi expert consensus studies to develop suicide first aid guidelines were available for English-speaking countries [5], Philippines [16], India [14], Japan [15], Sri Lanka [17] and Refugees and Immigrants [20]. These studies were conducted by research teams that included one or more of the authors. The studies are summarized in Table 1. All of these Delphi studies involved a panel of professional experts. In addition, the English-speaking Delphi study included a panel of consumers and the Refugee and Immigrant study included a panel of consumer advocates (who partially overlapped with its professional panel). In all of these studies, items were presented and rated in English for how important they were to include in the guidelines. The instructions were: “Please rate how important (from essential to should not be included) you think it is that each statement be included in the final guidelines”. The rating scale for each item had the following options: Essential, Important, Don’t know/depends, Unimportant, Should not be included. An item was regarded as endorsed by a panelist if it was rated as ‘essential’ or ‘important’ to include. Copies of the helping-action statements (items) included in each cultural Delphi study, as well as their endorsement ratings, were obtained by downloading relevant additional files from the publishing journal’s website (English-speaking, Philippines, India, Japan) or by contacting authors directly (Sri Lanka, Refugee and Immigrant). Some of the Delphi studies included items on suicide warning signs appropriate to the particular culture. These items were not included in the current analyses, which focus on suicide first aid actions.

**Table 1:** Characteristics of the Delphi studies

Study	Size of panels	Number of items rated	Number of items endorsed
English-speaking countries	41 professionals	436	164
	35 consumers		
Sri Lanka	14 professionals	531	304
Japan	32 professionals	176	56
India	30 professionals	168	71
Philippines	34 professionals	186	102
Refugee and Immigrant	34 professionals^a^	553	345
	17 consumer advocates^a^		

^a^7 participants belonged to both panels

Because the Delphi studies involved multiple rounds of rating, we used the ratings for each item on the round where its outcome was decided, e.g. if an item was re-rated in Round 2, we used the data from that round. Identical items across the different sets of Delphi items were matched, with corresponding endorsement ratings compiled into one data file. Items were matched using the search function in Microsoft Excel to locate novel words in item text. Researchers’ prior knowledge of guidelines content was also used in matching items with slightly different wording but similar meaning.

All items deemed similar by one of the authors (AMR) were discussed with another author (AFJ) to determine if they were similar enough for endorsement ratings to be comparable, or whether they are too dissimilar in meaning and need to be treated as separate items.

Some items that comprised two sentences sometimes appeared in some of the cultural Delphi studies as two separate statements, split at the full-stop. For items that were split and received similar endorsement ratings between them, the two ratings for the split item were averaged and included as an endorsement rating for the non-split item. For split items with dissimilar endorsement ratings, the two ratings for the split item were treated as different items to the original two-sentence non-split statement.

### Data analysis

To quantify agreement across a pair of expert panels, the percentage endorsement for each item by a panel was calculated and a Pearson correlation of item endorsement rates computed between the panels. This method has been previously used to compare item endorsement rates of multiple expert panels (e.g. professional, consumer advocate, carer advocate) within Delphi studies [1]. A correlation matrix was developed using data from all available Delphi studies. Because the Delphi studies had varying number of items in common, the number of items that each correlation was based on varied between pairs of studies from 85 (English-speaking country professionals with Japan professionals) to 542 (Refugee and Immigrant professionals with Refugee and Immigrant consumer advocates).

A missing values analysis showed that data on only 74 items were available across all Delphi panels. In order to include a greater number of items that were common across most of the Delphi panels, multiple imputation was carried out. Values were imputed for items that were missing ratings from up to 2 of the 8 Delphi panels using the Fully Conditional Specification algorithm (utilising the multiple chain method), with imputed values constrained between 0 to 100. This allowed a correlation matrix to be computed for 109 items across all panels. Data were analysed using SPSS v23.

To explore areas of difference between panels, an analysis was carried out of items that were endorsed by at least 80% of one panel, but another panel was 30% or more lower in endorsement. A difference of 30% has been described as a ‘large’ effect size [21]. This analysis was carried out for the items that were common across all Delphi studies. Because there are many comparisons involved, interpretation has been focused on areas of consistency where one panel had large differences from three or more other panels.

## Results

Table 2 shows the 18 items that were endorsed across all the 8 Delphi panels. The percentage panel agreement required for an item to be endorsed was 80% rating it as ‘essential’ or ‘important’ for all panels, except for the Japanese one, which had an endorsement cutoff of 70% because of the greater use of ‘don’t know/depends’ by that panel. Table 3 shows additional items to those in Table 2 that were endorsed by the panels from the three lower-middle income countries.

**Table 2:** Items endorsed across all Delphi studies

The first aider should be able to recognise the warning signs of suicide.	
The first aider should appear calm and confident in the face of the suicide crisis, as this may have a reassuring effect for the suicidal person.	
The first aider should allow the suicidal person to discuss their feelings. A suicidal person may feel relief at being able to do so.	
The first aider should take all thoughts of suicide seriously. The lack of a plan for suicide is not sufficient to ensure safety.	
The first aider should ask the suicidal person if they have a plan for suicide.	
The first aider should ask the suicidal person if they have decided when they will carry out their plan.	
The first aider should find out if the suicidal person has already taken steps to secure the means to end their life.	
The first aider should ask the suicidal person if they have been using drugs or alcohol.	
The first aider should ask the suicidal person if they have ever made a suicide attempt in the past.	
The first aider should work collaboratively with the suicidal person to ensure their safety, rather than acting alone to prevent suicide.	
The first aider must keep in mind that they may not be successful in preventing suicide.	
The first aider should tell the suicidal person they care and want to help.	
The first aider should express empathy for the suicidal person.	
Suicidal thoughts are often a plea for help and a desperate attempt to escape from problems and distressing feelings. The first aider should therefore allow the suicidal person to talk about those thoughts and feelings.	
The first aider needs to allow the suicidal person to talk about their reasons for wanting to die.	
The first aider should find out what has supported the suicidal person in the past and whether these supports are still available.	
Safety plans should include 24-h safety contacts in case the suicidal person feels unable to continue with the agreement not to attempt suicide (such as a suicide helpline, professional helper or family member).	
The first aider should treat the suicidal person with respect and involve them in decisions about who else knows about the suicidal crisis.

**Table 3:** Additional items endorsed across the Delphi panels from the three lower-middle-income countries

If the first aider thinks someone might be having suicidal thoughts, they should ask that person directly.	
The first aider should not avoid using the word ‘suicide’. It is important to discuss the issue directly, without dread or expressing negative judgement.	
If the first aider clearly states that thoughts of suicide may be associated with a treatable disorder, this may instil a sense of hope for the suicidal person.	
The first aider should establish whether the person has definite plans and intentions to take their life as opposed to vague suicidal notions such as “what’s the point?” or “I can’t be bothered going on”.	
If the suicidal person is psychotic, the first aider should call a doctor, psychiatrist or other professional right away for the suicidal person.	
The first aider should remind the suicidal person that suicidal thoughts need not be acted on.	
The first aider should not argue or debate with the person about their thoughts of suicide.	
The first aider should encourage the suicidal person to do most of the talking.	
The first aider should discuss the ‘good things’ in a person’s life, their hopes for the future, and other reasons to live.	
The first aider should encourage the suicidal person to think about their personal strengths and the positive things in their life.	
By discussing specific problems, the first aider can help the person work out ways of dealing with the difficulties that seem insurmountable.	
The first aider should make sure any potentially harmful items are not available to the suicidal person by removing access to these items.	
The first aider should try to determine whether there is anything important in the person’s life which may reduce the immediate risk of suicide (e.g. attachments to children).	
The first aider should ask for help from the person’s relatives, friends or housemates to ensure the person does not have access to weapons, poisons, or other means for suicide.	
(When passing time during the crisis) It is preferable that the suicidal person chooses an activity which has been found in the past to help them to cope or that they enjoy.	

Table 4 shows Pearson correlations across items for the various panels based on complete data (upper diagonal) and based on imputed data (lower diagonal). Correlations were all .60 or above, indicating broad agreement in priorities for inclusion in guidelines. The correlations were consistently highest (all .8 or above) between the English-speaking panels and the Refugee and Immigrant panels. The Asian country panels tended to show somewhat lower agreement with each other, with correlations in the range .67 to .87. The lowest agreement was between the English-speaking panels on the one hand and the Asian country panels on the other, with correlations in the range .60 to .79.

**Table 4:** Correlations of endorsement rates across items for the Delphi panels

Expert panel	English-speaking country professionals	English-speaking country consumer advocates	Sri Lanka professionals	Japan professionals	India professionals	Philippines professionals	Refugee and immigrant professionals	Refugee and immigrant advocates
English-speaking country professionals	1.00	.92	.79	.71	.66	.68	.91	.84
English-speaking country consumers	.92	1.00	.76	.70	.64	.65	.88	.84
Sri Lanka professionals	.76	.69	1.00	.69	.75	.67	.82	.80
Japan professionals	.68	.67	.69	1.00	.80	.78	.72	.66
India professionals	.65	.60	.76	.78	1.00	.87	.70	.65
Philippines professionals	.69	.63	.68	.74	.87	1.00	.70	.65
Refugee and immigrant professionals	.91	.88	.87	.71	.71	.70	1.00	.91
Refugee and immigrant consumers	.85	.85	.82	.64	.66	.65	.92	1.00

The correlations from complete data are based on varying numbers of items depending on how many were common to the pair of Delphi studies. These varied from 85 to 542. The correlations from imputed data are based on 109 items that had no more than 2 missing values across Delphi panels

Correlations based on complete data are in the upper diagonal and correlations based on imputed data are in the lower diagonal

Table 5 shows the items that were consistently more likely or less likely to be endorsed by one panel compared to others. It is difficult to distinguish consistent themes from these differences without knowing the panel members’ reasons for the ratings they made. However, a few themes are apparent. English-speaking panelists were less likely to endorse attempting to dissuade the person from suicide (e.g. by discussing the morality of suicide, telling them how much they would be missed and giving hope of the benefits of treatment). The Japan panel was also less likely to endorse dissuading actions. English-speaking panelists and the Japan panel were also less likely to endorse working with the person to deal with specific problems in their life than were panelists from the lower-middle-income countries or the immigrant and refugee panels. Something else that stands out in examining these differences is that the Japan panel was less likely to endorse a number of items, but there were no items it was more likely to endorse, whereas Philippines, India and Sri Lanka showed the opposite pattern.

**Table 5:** Items showing large and consistent differences between panels^a^

English-speaking professional panel	
More likely to endorse:	
• The first aider should be aware that if a person is not suicidal, asking them cannot put the idea of suicide in their head.	
• The first aider should not discuss with the person whether suicide is right or wrong.	
Less likely to endorse:	
• If the first aider clearly states that thoughts of suicide may be associated with a treatable disorder, this may instil a sense of hope for the suicidal person.	
• The first aider should remind the suicidal person that they are loved and would be missed.	
• By discussing specific problems, the first aider can help the person work out ways of dealing with the difficulties that seem insurmountable.	
English-speaking consumer panel	
More likely to endorse:	
• The first aider should be aware that if a person is not suicidal, asking them cannot put the idea of suicide in their head.	
• The first aider should not discuss with the person whether suicide is right or wrong.	
Less likely to endorse:	
• If the first aider clearly states that thoughts of suicide may be associated with a treatable disorder, this may instil a sense of hope for the suicidal person.	
• By discussing specific problems, the first aider can help the person work out ways of dealing with the difficulties that seem insurmountable.	
Philippines panel	
More likely to endorse:	
• If the person is using drugs or alcohol, the first aider may not be able to believe them if they say they are not suicidal.	
• The first aider should ask the suicidal person if they have ever known anyone who has died by suicide.	
• The first aider does not need to be with the suicidal person all the time, but should check on them regularly.	
• If the person is suicidal, the first aider should call a doctor, psychiatrist or other professional right away for the suicidal person.	
• If the suicidal person has a weapon, the first aider should try to take it away from them.	
• The fact that the suicidal person is still alive, and talking to the first aider about their feelings, means that they are not quite sure about suicide. The first aider should point this out as a positive thing.	
• By discussing specific problems, the first aider can help the person work out ways of dealing with the difficulties that seem insurmountable.	
• If the suicidal person agrees to hand over the means of suicide, on the condition that they can have them back if they want them, the first aider should agree to this.	
India panel	
More likely to endorse:	
• If the first aider clearly states that thoughts of suicide may be associated with a treatable disorder, this may instil a sense of hope for the suicidal person.	
• The first aider should reassure the suicidal person that they understand how badly they feel.	
• By discussing specific problems, the first aider can help the person work out ways of dealing with the difficulties that seem insurmountable.	
• If the suicidal person agrees to hand over the means of suicide, on the condition that they can have them back if they want them, the first aider should agree to this.	
Sri Lanka panel	
More likely to endorse:	
• If the first aider clearly states that thoughts of suicide may be associated with a treatable disorder, this may instil a sense of hope for the suicidal person.	
• The first aider should ask the suicidal person if they have ever known anyone who has died by suicide.	
• The first aider should reassure the suicidal person that they understand how badly they feel.	
• By discussing specific problems, the first aider can help the person work out ways of dealing with the difficulties that seem insurmountable.	
• The first aider should encourage the suicidal person to consider the consequences of suiciding, especially the effect it may have on the people they care about.	
Japan panel	
Less likely to endorse:	
• The first aider should establish whether the person has definite plans and intentions to take their life as opposed to vague suicidal notions such as “what’s the point?” or “I can’t be bothered going on.”	
• If the suicidal person is psychotic, the first aider should: Call a doctor, psychiatrist or other professional right away for the suicidal person.	
• The first aider should remind the suicidal person that suicidal thoughts need not be acted on.	
• The first aider should not discuss with the person whether suicide is right or wrong.	
• The first aider should encourage the suicidal person to do most of the talking.	
• The first aider should discuss the ‘good things’ in a person’s life, their hopes for the future, and other reasons to live.	
• The first aider should encourage the suicidal person to think about their personal strengths and the positive things in their life.	
• By discussing specific problems, the first aider can help the person work out ways of dealing with the difficulties that seem insurmountable.	
Immigrant & refugee professional panel	
More likely to endorse:	
• The first aider should be aware that if a person is not suicidal, asking them cannot put the idea of suicide in their head.	
• By discussing specific problems, the first aider can help the person work out ways of dealing with the difficulties that seem insurmountable.	
Immigrant & refugee consumer panel	
More likely to endorse:	
• By discussing specific problems, the first aider can help the person work out ways of dealing with the difficulties that seem insurmountable.	

^a^Items endorsed by one panel (80%+) but with three or more other panels showing large differences (30%+)

## Discussion

Comparison of items endorsed across all Delphi studies shows a number of actions that are recommended across a broad range of countries and cultures. These cover assessing the risk of suicide, listening to the person, showing care and respect, and ensuring the person’s safety. A notable omission from these items is recommendations to refer the person to professional help. This omission may reflect the fact that items about professional help-seeking were not consistently included across the various Delphi questionnaires. It might also reflect limitations in the availability or feasibility of professional help in some countries, where services like suicide hotlines are not always available and mental health services can be scarce, particularly in rural areas. Thus, the list of items that were endorsed across studies should be regarded as a minimal cross-cultural consensus rather than exhaustive.

We also specifically examined actions that were endorsed across the three lower-middle-income countries (India, Philippines and Sri Lanka), which resulted in a larger pool of common items, indicating the feasibility of developing guidelines that might be generalizable to a broader group of low- and middle-income countries. Although additional items were endorsed in the three lower-middle-income countries, these may not necessarily reflect cultural differences between these countries and the rest. It may instead be due to the fact that it is easier to get consistent endorsement across three expert panels than across eight panels.

Despite these commonalities, there were also a number of differences between panels For example, the English-speaking and Japan panels were less likely to endorse attempting to dissuade the person from suicide or attempt to solve their problems than were panelists from the lower-middle-income countries or the immigrant and refugee panels. This could partially be explained by the fact that the lower-middle income countries in these studies are generally considered collectivistic societies, i.e. people are expected to look after each other (e.g. helping to solve one’s problems). Furthermore, in English-speaking countries and more ‘westernized’ societies such as Japan the bio-medical model of suicide, which attributes suicidal behavior to mental illness, is widespread and as a consequence an individual might see him/herself as unable to ‘solve the problem’, as this requires the intervention of a mental health professional or other health/medical professional [13]. There were also differences between panels in endorsement rates that may reflect variation in willingness of panel member to state firm views on the items. Cultural differences were also reported in the original Delphi studies. For example, in the Filipino study it was noted that religious and spiritual concepts appear to be more important in suicide prevention than in India or Japan [16]. By contrast, in the Japanese study it was noted that the experts were less likely to endorse actions that involved a closer personal distance and that some first aid strategies highly endorsed in other countries might be seen as socially inappropriate in Japan [15]. In the Refugee and Immigrant study, it was noted that refugees and immigrants sometimes have a fear and distrust about emergency services and that females from some cultural backgrounds may not be permitted to make decisions regarding their own health alone [20]. Availability of services is another culturally varying factor. For example, in the Sri Lankan study the lack of services in rural areas was noted [17].

Developing separate suicide first aid guidelines for each cultural group is the ideal. As well as providing specific cultural tailoring, it provides buy-in from the target cultural group by involving relevant experts at an early stage. However, to do this for every country, including for cultural minority groups within a country, is very resource intensive. It can also be difficult to recruit lived-experience experts from cultural groups that lack consumer advocacy organizations, which was the case with the Asian Delphi studies included here.

The alternative is to develop guidelines based on the consensus of experts from a broader range of cultural groups, including lived-experience experts where they are available. These broader guidelines may then be supplemented by expert consensus guidelines on additional cultural considerations that are specific to the target group. For example, this approach has been used to develop guidelines on additional considerations when giving mental health first aid (which includes suicide first aid) to Aboriginal and Torres Strait Islander adolescents [22], Iraqi refugees [23] and LGBTIQ people [24]. These additional cultural considerations have then been incorporated in Mental Health First Aid training [7, 9].

## Conclusions

While this study has involved only suicide first aid guidelines, the findings may have implications for the broader development of mental health first aid guidelines for assisting a person developing a mental health problem or in a mental health crisis [25–33]. While previous research has sought the expertise of professionals, consumer advocates and carer advocates from developed English-speaking countries, it may be feasible to develop mental health first aid guidelines that have broader applicability, for example to high-income countries or to low- and middle-income countries, which can then be supplemented by consensus on local cultural and health system considerations.

## Abbreviations

LGBTIQ: Lesbian, Gay, Bisexual, Transgender, Intersex and Questioning
SPSS: Statistical Package for the Social Sciences

## References

[1] Jorm AF (2015). Using the Delphi expert consensus method in mental health research. Aust N Z J Psychiatry.

[2] Kelly CM, Jorm AF, Kitchener BA, Langlands RL (2008). Development of mental health first aid guidelines for suicidal ideation and behaviour: a Delphi study. BMC Psychiatry.

[3] Jorm AF, Kitchener BA (2011). Noting a landmark achievement: mental health first aid training reaches 1% of Australian adults. Aust N Z J Psychiatry.

[4] Hart LM, Jorm AF, Paxton SJ, Cvetkovski S (2012). Mental health first aid guidelines: an evaluation of impact following download from the world wide web. Early Interv Psychiatry.

[5] Ross AM, Kelly CM, Jorm AF. Re-development of mental health first aid guidelines for suicidal ideation and behaviour: a Delphi study. BMC Psychiatry. 2014;14(1) 10.1186/s12888-014-0241-8 PMC4199061 25213799

[6] Kelly CM, Kitchener BA, Jorm AF (2013). Youth mental health first aid: a manual for adults assisting young people.

[7] Kelly CM, Kitchener BA, Jorm AF (2017). Youth mental health first aid: a manual for adults assisting young people.

[8] Kitchener BA, Jorm AF, Kelly CM (2013). Mental health first aid manual.

[9] Kitchener BA, Jorm AF, Kelly CM (2017). Mental health first aid manual.

[10] Kelly CM, Blee FL, Claessen GH (2016). Mental health first aid for the suicidal person: course handbook.

[11] General Practice Mental Health Standards Collaboration (GPMHSC) (2016). Suicide prevention and first aid: a resource for GPs.

[12] American Psychiatric Association. American Psychiatric Association practice guidelines for the treatment of psychiatric disorders: compendium. Arlington: American Psychiatric Association; 2006.

[13] Colucci E, Lester D (2013). Suicide and culture: understanding the context.

[14] Colucci E, Kelly CM, Minas H, Jorm AF, Chatterjee S (2010). Mental health first aid guidelines for helping a suicidal person: a Delphi consensus study in India. Int J Ment Health Syst.

[15] Colucci E, Kelly CM, Minas H, Jorm AF, Suzuki Y (2011). Mental health first aid guidelines for helping a suicidal person: a Delphi consensus study in Japan. Int J Ment Health Syst.

[16] Colucci E, Kelly CM, Minas H, Jorm AF, Nadera D. Mental health first aid guidelines for helping a suicidal person: a Delphi consensus study in the Philippines. Int J Ment Health Syst. 2010;4 10.1186/1752-4458-4-32 PMC3017011 21167076

[17] De Silva SA, Colucci E, Mendis J, Kelly CM, Jorm AF, Minas H. Suicide first aid guidelines for Sri Lanka: a Delphi consensus study. Int J Ment Health Syst. 2016;10(1) 10.1186/s13033-016-0085-3 PMC5004258 27579055

[18] Colucci E, Heredia Montesinos A (2013). Violence against women and suicide in the context of migration. Suicidology Online.

[19] Colucci E, Too T, Bergen DV, Montesinos AH, Gottingen S-OM (2014). Culture, cultural meaning(s) and suicide among immigrants and refugees. Suicide in the context of migration. Edn.

[20] Colucci E, Jorm AF, Kelly CM, Minas H (2017). Suicide first aid guidelines for a person from immigrant or refugee background: a Delphi consensus study.

[21] Rosenthal JA (1996). Qualitative descriptors of strength of association and effect size. J Soc Serv Res.

[22] Chalmers KJ, Bond KS, Jorm AF, Kelly CM, Kitchener BA, Williams-Tchen A (2014). Providing culturally appropriate mental health first aid to an aboriginal or Torres Strait islander adolescent: development of expert consensus guidelines. Int J Ment Health Syst.

[23] Uribe Guajardo MG, Slewa-Younan S, Santalucia Y, Jorm AF (2016). Important considerations when providing mental health first aid to Iraqi refugees in Australia: a Delphi study. Int J Ment Health Syst.

[24] Bond KS, Jorm AF, Kelly CM, Kitchener BA, Morris SL, Mason RJ (2017). Considerations when providing mental health first aid to an LGBTIQ person: a Delphi study. Advances in Mental Health.

[25] Bond KS, Jorm AF, Kitchener BA, Kelly CM, Chalmers KJ (2016). Development of guidelines for family and non-professional helpers on assisting an older person who is developing cognitive impairment or has dementia: a Delphi expert consensus study. BMC Geriatr.

[26] Bond KS, Jorm AF, Miller HE, Rodda SN, Reavley NJ, Kelly CM, Kitchener BA (2016). How a concerned family member, friend or member of the public can help someone with gambling problems: a Delphi consensus study. BMC Psychol.

[27] Hart LM, Jorm AF, Paxton SJ, Kelly CM, Kitchener BA (2009). First aid for eating disorders. Eating Disord.

[28] Kelly CM, Jorm AF, Kitchener BA (2009). Development of mental health first aid guidelines for panic attacks: a Delphi study. BMC Psychiatry.

[29] Kelly CM, Jorm AF, Kitchener BA. Development of mental health first aid guidelines on how a member of the public can support a person affected by a traumatic event: a Delphi study. BMC Psychiatry. 2010;10 10.1186/1471-244X-10-49 PMC2904289 20565918

[30] Kingston AH, Jorm AF, Kitchener BA, Hides L, Kelly CM, Morgan AJ, Hart LM, Lubman DI (2009). Helping someone with problem drinking: mental health first aid guidelines - a Delphi expert consensus study. BMC Psychiatry.

[31] Kingston AH, Morgan AJ, Jorm AF, Hal K, Hart LM, Kelly CM, Lubman DI (2011). Helping someone with problem drug use: a delphi consensus study of consumers, carers, and clinicians. BMC Psychiatry.

[32] Langlands RL, Jorm AF, Kelly CM, Kitchener BA (2008). First aid recommendations for psychosis: using the Delphi method to gain consensus between mental health consumers, carers, and clinicians. Schizophr Bull.

[33] Langlands RL, Jorm AF, Kelly CM, Kitchener BA (2008). First aid for depression: a Delphi consensus study with consumers, carers and clinicians. J Affect Disord.

